# Land Cover Change and Its Impact in Crop Yield: A Case Study from Western Nepal

**DOI:** 10.1155/2022/5129423

**Published:** 2022-02-21

**Authors:** Ajay Bhandari, Rajeev Joshi, Mahendra Singh Thapa, Ram Prasad Sharma, Sumit Kumar Rauniyar

**Affiliations:** ^1^Tribhuvan University, Institute of Forestry, Pokhara Campus, Post Box No. 43, Hariyokharka 15, Pokhara, Gandaki Province, Nepal; ^2^Faculty of Forestry, Agriculture and Forestry University, Hetauda−44107, Makawanpur, Nepal; ^3^Amity Global Education (Lord Buddha College), CTEVT, Tokha-11, Kathmandu−44600, Nepal; ^4^Tribhuvan University, Institute of Forestry, Office of the Dean, Kirtipur, Kathmandu, Bagmati Province, Nepal

## Abstract

This study was conducted in Tanahun district of Gandaki Province, Nepal, to analyze the land cover change over two decades, the migration effect in land cover, and the impact caused in crop production by *Rhesus macaque*. Landsat TM/ETM+ for land use of 2000 and 2010 extracted by ICIMOD and Landsat 8 OLI/TIRS satellite images for land cover 2019 were downloaded from the USGS website. A purposive sample for household survey was carried out based on crops damaged by the monkey. Two hundred and fifty households were taken as samples. The Landsat images were analyzed by ArcGIS, and the social data were analyzed using SPSS and MS Excel. Land cover change data revealed increment of forest cover from 36.57% to 40.91% and drastic decrease in agriculture crops from 57.52% to 43.78% in the period of 20 years. The accuracy of the data showed overall classification accuracy of 86.11%, 81.08%, and 75% with overall kappa statistics 0.83, 0.77, and 0.74, respectively. The migration effect in the land cover was related to remittance and migrated members and found a significant positive relationship. Analyzing the trend of production with an increase in the forest cover, 21% decrease in paddy, 5% decrease in maize, and 26% decrease in millet were found as compared to the production in 2000. The econometric model concluded that the quantity of crop damage was negatively significant in relation to distance from forest and distance from water body while positively significant to distance from settlements and distance from owner's home. The quantity of crop damage was estimated 113.89 kg per household, and the cost was 78.82 USD. This study recommends active forest management; regular thinning, and weeding. Remittance generated should be invested in the agriculture field by the households. Damage relief should be made available for the damage cost by *Rhesus macaque*.

## 1. Introduction

Nepal, being a landlocked mountainous country, has an intense level of cultural and biological diversities [1]. For the protection of biological diversity, Nepal has covered 23.23% of the land as a protected area [2]. Despite the protection of biological and cultural diversities, agricultural land has provisioning services to maintain food security [3]. Land is being a finite natural national resource, and efficient management is vital for the economic growth and development of the country [4]. The mountain regions are found more sensitive to land use and land cover changes (LULC) [5]. LULC are distinct yet closely linked characteristics of the earth's surface [6]. Generally, the mountain region experiences the impacts of even small changes more strongly than plain areas [7]. These impacts are not confined solely to the mountain areas where the change occurs but is also transmitted to lowland areas where the impacts are intensified due to the steep gradients of the mountain slopes [8]. The term land cover originally referred to the kind and state of vegetation, such as forest or grass cover, but it has broadened in subsequent usage to include other things such as human structures, soil types, biodiversity, surface, and groundwater [9, 10]. Land cover categories could be cropland, forest, wetland, pasture, roads, and urban areas among many others. Among concerns about global environmental change, some issues related to LULC and its change over time is becoming increasingly recognized [11]. The process of cropland change is complex and occurs over different pathways, with a diversity of magnitudes and rates [12]. It is always dynamic and occurs differently when observed at different scales [13]. In historical cropland sectors, several studies have been well documented and have created long-term spatial datasets detailing the overall changes in cropland [14].

Land cover change is a common phenomenon that is demand-driven by the locals residing around it [15]. The study on LULC change from the Koshi basin in Nepal also shows the population as a major driver of expanding agricultural land in the last three decades [16]. Many studies found the connection between species loss and the quality (size, composition, and structure) of a forest. Gascon et al. and Lin et al. reported a strong negative correlation between forest size, density, structure, and quality to the number of species using it [17, 18]. Naughton et al. [19], Hoare [20], and Roy [21] described human population growth, land use transformation, species, and their habitat loss, fragmentation, development, ecotourism, increasing livestock population, competitive exclusion of wild herbivores, abundance, and distribution of wild prey and increasing wildlife population as sources of conflicts [19–21]. There are various drivers of land cover change. Drivers may include development activities such as the construction of infrastructures, settlement, and other demographic processes like migration [22]. Migration refers to the movement of the people from or to the place residing [23]. Labor migration either men and/or women eager to work abroad results in large amounts of remittances which can increase the foreign development aid in the value [24]. Many mountain countries such as Kyrgyzstan, Tajikistan, Nepal, Lesotho, or Armenia experience migration towards the lowlands, urbanized areas, and abroad [25]. These countries, largely affected by these phenomena, are among the highest recipients of remittances [26]. There are several causes of migration. Poverty, lack of employment opportunities, education, and structural inequalities are the push factors, and better economic opportunities are the pull factors [27]. The combination of these factors leads to the land cover change. The cropland has been deduced to partial land due to the effect of migration. This has led to a humungous decrease in crop yield. Furthermore, the increase of barren land has produced an increase in forest cover. Thus, there are human-wildlife conflicts, between monkeys and crops, wild bears and crops, and leopards and livestock. Crop depredation by the monkey has been serious all around the country. Thus, on the verge of inclusion of damage relief caused by the monkey, its damage cost and precaution are deeply required.

## 2. Materials and Methods

**Figure 1:**
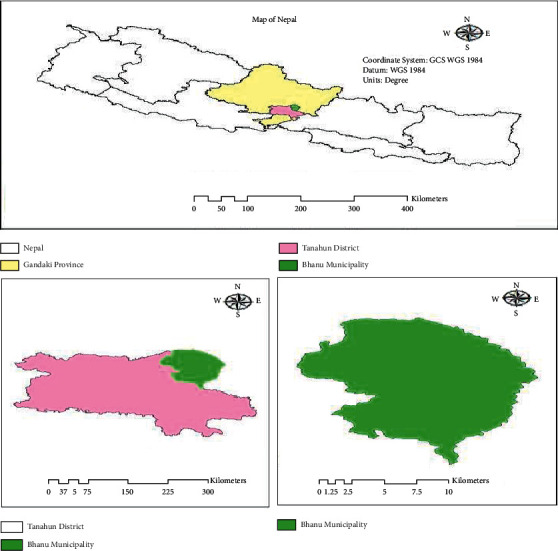
Map of Nepal showing the study area.

This study was conducted in the Bhanu Municipality of Tanahun district of Nepal (Figure 1). Bhanu Municipality is in the Gandaki zone of Tanahun district which is one of the 11 districts of Gandaki Province. It is situated 140 km west of Kathmandu, the capital city of Nepal, and 68 km East of Pokhara, the headquarters of Gandaki Province. Bhanu Municipality is surrounded by Gorkha and Lamjung districts in the east, Byas Municipality in the west, Lamjung district in the north, and Bandipur Rural Municipality and Gorkha district in the south direction. Geographically, the municipality is extended from 84°18′37″ to 84°28′39″ east longitude and 27°57′07″ to 28°47′31″ north latitude.

The main data in the study included temporal satellite data of Landsat TM/ETM + for land use of 2000 and 2010 extracted by ICIMOD and OLI/TIRS (operational land imager/thermal infrared sensor) for the year 2019 (20 years with 10 years interval) for mapping (Table 1). Topographic maps of 1 : 25,000 scale and digital topographic data with contour intervals of 20 m published by the Survey Department, Government of Nepal (GoN), was used as ancillary data.

250 households were taken purposively as a sample for the semistructured questionnaire survey on the basis of crop attacks by wildlife. Primary data were collected to assess the socioeconomic condition of the households. Secondary data like the status of community forest, population statistics, and agriculture production of cereal crops were obtained from Division Forest Office, Central Bureau Statistics and Department of Agriculture. The recall method was used to measure productivity among the periods over time. The respondents were asked to recall their past respond to productivity and land cover change among different years. The community meeting was conducted to obtain general information about the status of crop damage by monkeys, bears, and other wild animals and the local rate of agricultural products. A structured questionnaire was designed and used to gather information about the amount of crop damage and respondents' perception towards monkey conservation and compensation scheme.

ArcGIS 10.5 was used for estimation; mention the classification method with an algorithm, classification accuracy, and change detection of land cover change. The confusion matrix was performed by comparing error values for each class that was classified with its respective value in the ground truth data. For the image 2000 and 2010, Google earth points were used as ground truth points (GTPs), and for 2019 image data, GTPs were collected from the study area. Total GTP for the accuracy assessment was 75 for land cover 2000 and 2010, while 80 GTPs were used for land cover 2019.

The kappa coefficient (*K*^) was then calculated using the following equation:

(1) $$\begin{array}{c} K\wedge =\frac{N\sum_{i=1}^{r}X_{ii}-\sum_{i=1}^{r}R+C}{N^{2}-\sum_{i=1}^{r}R+C}, \end{array}$$

where *r* represents the number of rows/columns in confusion matrix, *X*_*ii*_ represents the number of observation in row *i* and column *i*, *R* represents the total number of row *i*; *C* represents the total number of column *i*, and *N* represents the number of observations.

The kappa statistics provide a statistically valid assessment of the quality of classification and was used to assess overall class accuracy.

Variables were generated from the questionnaire survey, and regression analysis was carried out using SPSS for the variables that are responsible for the land cover change.

The econometric model developed by Hill [28], Hill [29], and Bayani [30] was tested and used to calculate the amount of crop damage [28–30].

(2) $$\begin{array}{c} Y=C+\beta_{1}.X_{1}+\beta_{2}X_{2}+\beta_{3}X_{3}+\beta_{4}X_{4}, \end{array}$$

where *Y* represents the amount of crop damage in kg, *C* represents the constant, *X*_1_ represents the distance from water sources (meter) 1, *X*_2_ represents the distance from respondents' home (meter) 2, *X*_3_ represents the distance from nearby settlement (meter) 3; *X*_4_ represents the distance from forest (meter) 4, *ß*_1_, *ß*_2_, *ß*_3_, and *ß*_4_ are the coefficients associated with these 1, 2, 3, and 4 parameters, which denote the marginal effect of the change in the corresponding parameter to the amount of crop damaged. Cost of crop damage per household was calculated by using the following formula:

(3) $$\begin{array}{c} \text{Cost of image}=\frac{\sum_{i=1}^{n}\sum_{j=1}^{n}q_{ij}\times p_{ij}}{N}, \end{array}$$

(Adopted from Poudel and Shrestha) [31],where *q* represents the quantity of crop damaged in kg, *p* represents the price of crop damaged in USD, *i* represents the varieties of crops, *j* represents the households, and *N* represents the total number of households.

## 3. Result and Discussion

### 3.1. Land Cover Change

**Figure 2:**
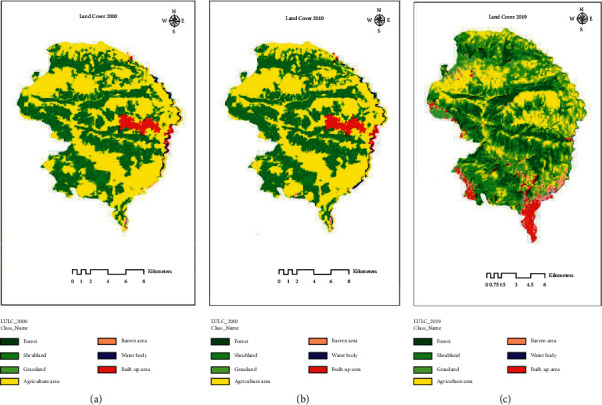
Land cover change in different periods. (a) Land cover 2000. (b) Land cover 2010. (c) Land cover 2019.

The land cover change in different periods is shown in Figure 2. For this, supervised classification was used, and seven land cover classes, forest, shrubland, agriculture area, grassland, barren land, waterbody, and built-up area, were identified. The results from Figures 2(a)–2(c) show the change in the land cover in the periods of two decades. The change in the cover from different classes can be viewed. The results given in Table 2 show the drastic change from the periods 2010 to 2019, whereas quite similar from 2000 to 2010 A.D. The change in forest cover is increasing at a steady rate. Forest cover has increased from 36.57% to 40.91% (988 ha) in the period of 20 years. The increase in shrub cover is seen abundantly between periods 2010 and 2019.

The agricultural land has drastically reduced from 57.52% to 43.78% (2452 ha), whereas barren land is seen increased from 0.08 to 4.10%. The results depicted here are different between Terai and Hill regions. The study conducted by Pariyar and Singh [32] in the Chitwan district showed that the agricultural land area in the district had shrunk by about 11% during the period 1978–1992, whereas 14.26% shrinkage is been seen in my study [32]. The study conducted by Chapagain et al. [33] in Panchthar also showed increase in the forest cover by 249 ha from 1994 to 2004 and loss in agriculture by 142 ha, whereas increment by 279 ha in forest class and loss in agriculture by 142 ha from 2004 to 2014 [33]. The abovementioned study was conducted in a small area (only Sidin VDC), while our study was conducted in the whole municipality. These all results show that the midhills land is dominated by forest which was previously dominated by agriculture.

### 3.2. Accuracy Assessment

Although viewing accuracy as a simple concept, it is a very difficult variable to assess and is associated with many problems [34]. The basic accuracy measure is the overall accuracy, which is calculated by dividing the correctly classified pixels by the total number of pixels checked [35]. Concerning the overall producer and user accuracy for the classified imagery classes during the study periods, the result revealed excellent user accuracy for nearly all the classes in all years. However, some classes recorded satisfactory user accuracy, e.g., the class of grassland in satellite image of 2010 (Table 2).

The results of supervised classification of land cover in Bhanu Municipality for the Landsat image 2000, 2010, and 2019 showed an overall classification accuracy of 86.11%, 81.08%, and 75% with overall kappa statistics 0.83, 0.77, and 0.74, respectively (Tables 2 and 3). This proves that the classification was within the excellent range [36]. The results of accuracy assessment of supervised classification of land cover classes in Bhanu Municipality in all years showed excellent classification for all classes, with exception of grassland class in the image Landsat TM 2010 and the class of barren land in the image 2010 which showed poor classification (Tables 4 and 5).

### 3.3. Change Matrix

**Figure 3:**
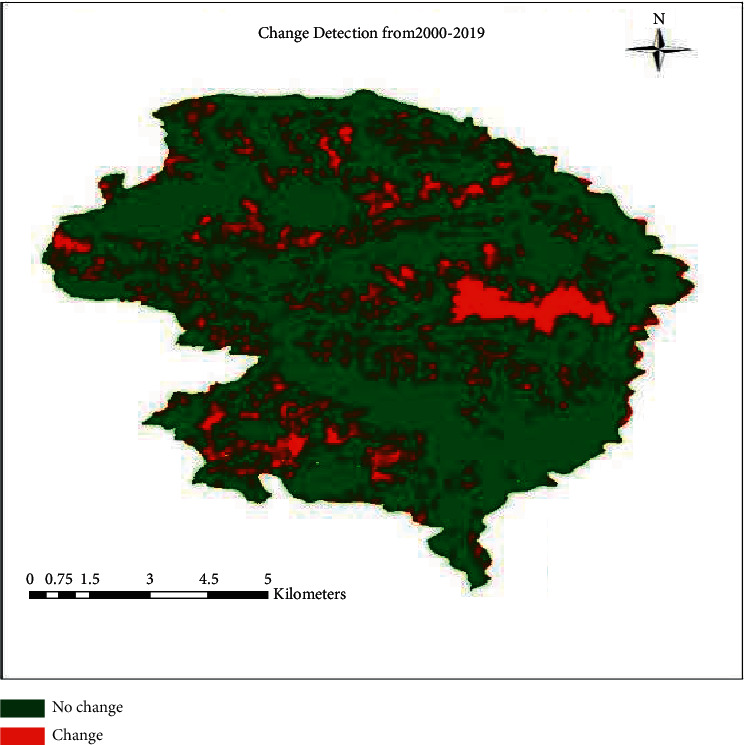
Land cover change detecting the migration effect in land cover.

The red color in Figure 3 indicates the change in the class, whereas the green color indicates no change in the study area. Land cover change matrix and change detection map give the evidence of change in class.

The results show the change of class from agriculture to other is 2064.3 ha, which is far more than from other classes. The change from others to barren is also increased to 750.42 ha, similarly is the case for other to shrub which is 826.81 ha (Table 4). The local perception is that the change in agricultural land is seen, and this is due to the conversion from agricultural land to barren land which is the effect of migration.

### 3.4. Migration Effect in Land Cover

The households (HHs) survey included 250 HHs. Out of 250 HHs, the total number of individuals in the family was 1693 with 7 average family sizes. The migrated member was found to be 649 out of which 337 was found to be migrated in cities of Nepal (domestic) and 312 was found to be migrated out of country (international) preferably in the Gulf countries excluding some in other Europe, USA, Australia, and India, approximately 1 out of 5 individuals. People migration towards the cities and abroad are mainly due to two reasons: first for the need of facilities to make the life easy and second for earning and helping their family economically.

Table 5 provides how the dependent variables, migrated member, and remittance are corelated with the independent variable barren land. The coefficient of independent variables (*β* = 0.499 and *β* = 0.331) has a positive significance with the independent variable (*p* < 0.05). This means the conversion of agricultural land into barren land is directly related to migrated members and remittance of the out migrated members.

This result suggests a positive effect of international migration on forest regeneration in Nepal, particularly in more agriculturally suitable areas. This study wants to give insight into mechanisms through which international migration affects reforestation. An example is being set that outmigration has significantly influenced the effect of international migration in increasing barren land that has mediated by reductions in agricultural activity at the household level, and the remittances generated from migration households invest to relocate entire households from rural to urban areas [37]. Also, evidence based on the study by Oldekop et al. [38] suggests households are not able to utilize remittances in the agriculture productivity by hiring the wage laborers which is also shown in my study that the remittances from the international migration have a positive effect on increasing the barren land which directly increases the forest cover [38]. The study conducted by Chapagain et al. [33] also concluded that agriculture labor shortage is a major factor of land-use change, and labor migration has resulted in agricultural labor shortage because of the people have transformed cereal crops into cash crops [33].

### 3.5. Agriculture Productivity with Change in Land Cover

**Figure 4:**
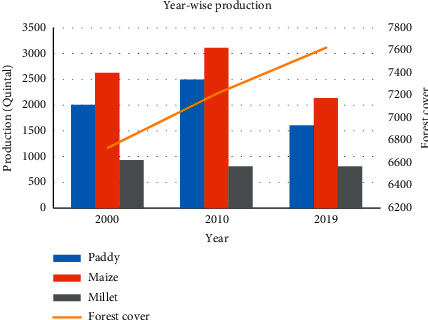
Productivity with an increase in forest cover.

Figure 4 shows the trend of production with a change in forest cover. The forest cover of the study area is increasing with the increase in barren land, and hence, the productivity of the agricultural crops is being decreased. The results show 21% decrease in paddy, 5% decrease in maize, and 26% decrease in millet compared to the production in 2000. The paddy and wheat production from 2000 to 2010 is seen increased which might be the result of a change in breed of the agricultural crops. The local breed of paddy was replaced by a hybrid breed (Ramdhan) during this period. The other reason for the increase in production might be due to the use of fertilizers and a low rate of migration. The reason for the decrease in production from 2010 to 2019 was due to the decrease in cropped (agriculture) area.

**Figure 5:**
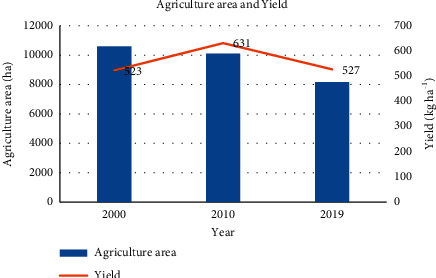
The graph showing agriculture area and yield obtained.

Comparing the agriculture area and productivity yield gives the best results. The yield was 523 kg ha^−1^ and 631 kg ha^−1^ in the year 2000 and 2010, respectively. Similarly, the yield was found 527 kg ha^−1^ in the year 2019 (Figure 5). The production of the study site is not only decreased but also the yield increased during the period of 2010 and 2019. The rise in temperature prolonged rainfall and use of an improved variety of crops, and application of the adequate fertilizer may also increase production/productivity. The labor shortage people have transformed cereal crops to cash crops, i.e., cardamom which requires less labor in comparison to cereal crops. Importantly, people have also transformed low productive land and in some cases good rain-fed terraces to the private forest and started getting cash by selling logs [33]. This finding can also be due to the increased rate of crop damage by wild animals.

### 3.6. Impact of *Rhesus macaque* in Crop Production

The variables used for crop damage were the distance from the settlement, distance from home, distance from water, and distance from forests.  Table 6 provides the coefficient for each variable. The variables distance from settlement and distance from home are positively significant (*p* < 0.05) with their *β*-coefficient 0.33 and 0.429, respectively, with crop damage, whereas distance from water and distance from the forest are negatively significant (*p* < 0.05) with *β*-coefficient 0.159 and 0.399, respectively. This means crop damage increases with the increase in distance from home and settlement and decreases in distance from forest and water. The land parcel near to forest and water has high crop damage by *Rhesus macaque*. The total crop damage for each HHs was found to be 113.89 kg per year.

### 3.7. Testing of the Econometric Model

The econometric model was used and tested for calculating the crop damage per kg per household. The following table shows the fitness of the econometric model (Table 7). The econometric model was found significant (*p* < 0.05) and strongly correlated with the variables of regression (*R* = 0.93 and *R*^2^ = 0.89).

### 3.8. Cost of Crop Damage

The individual household cost of crop damage was extracted using the formulae of data analysis. The annual crop damage amount in the studied area was found to be 113.89 kg per household per year that worth 78.82 USD per household. Crop damage is viewed as one of the common types of HWC which has a significant impact as compared to livelihoods. The amount of damage differs from place to place depending upon the periphery and intensity of the raiding. Ghimirey [39] determined that the cost of damaged crops was USD 726.02 per household per year in Makalu Barun National Park [39]. The economic value of the damage in my finding is less than one made by Ghimirey [39]. His study was concentrated on the periphery of the national park which is more affected than the other area, and hence, the cost of damage is higher than this finding. This finding on total damage also contrasts with the finding of the studies carried out in the protected areas (PAs). Annual crop damage by wildlife cost was equal to USD 210.02 per household nearby the Gaurishankar Conservation Area in Nepal [40]. The abovementioned study area was conducted in the periphery of PAs, but the similar study conducted by Shrestha and Poudel [31] in the nonprotected area resulted the damage cost of 75.10 USD per household with crop damage as 183.46 kg per household, and damage percent of crops by *Rhesus macaque* was 16.51% [31]. Although the damage amount (113.89 kg) is less and the cost (78.82 USD) is high in my study as compared to the study by Poudel and Shrestha [31], the local market rate of crops and the exchange rate was higher than their study [31]. Due to the higher intensity of crop raiding, the amount of damage is obviously higher around PAs in comparison to the damage that occurred in or around nonprotected areas.

## 4. Conclusion

The forest cover is increasing in the midhills region of Nepal, while agricultural land is being decreased. International migration plays as a key driver in the increase in the forest cover and decreases in agriculture making labor shortage in the midhills. The increase in the forest cover consequently resulted in an increase in the number of wildlife. Due to the increase in the number of wildlife in the study area, damage by *Rhesus macaque* has created a critical impact on the productivity of cereal crops. The migration effect in the land cover was related to remittance and migrated members and found a significant positive relationship. The econometric model concluded that the quantity of crop damage was negatively significant in relation to distance from forest and distance from water body while positively significant to distance from settlements and distance from owner's home. Finally, it can be concluded that the increase in forest cover has decreased agriculture production by decreasing the area. Also, increase in wildlife has affected in yield of agriculture by crop damage.

Active forest management like regular thinning and weeding should be done by the community forest users. To decrease the rate of migration, employment opportunities should be created within the country. The creation of water holes and plantation of wildlife food in the forest area is essential. Damage relief should be made available for the damage cost by *Rhesus macaque* which has added pressure in maintaining livelihoods. This study is conducted only based on two independent variables for relating with barren land. Other variables such as labor availability and fertilizers availability can be also used. Not only the crop damage in Bhanu Municipality but also leopard-human attack can also be the topic of research, where the conflict rate has been seen higher now-a-days.

## Figures and Tables

**Table 1:** Image characteristics of the satellite data.

Satellite images	Projection/datum	Resolution	No. of bands	Acquisition date
Landsat7_ETM	UTM/WGS 1984	30*∗*30	9	ICIMOD; February 2020
Landsat 8 OLI/TIRS	UTM/WGS 1984	30*∗*30	11	February 2020

**Table 2:** Accuracy assessment of different land cover classes.

Accuracy	Land cover 2000	Land cover 2010	Land cover 2019			
Class	User's accuracy	Producer's accuracy	User's accuracy	Producer's accuracy	User's accuracy	Producer's accuracy

Forest	93.75	83.33	82.35	82.35	70.00	82.35
Grassland	80.00	80.00	72.76	80.00	80.00	80.00
Agriculture	83.33	93.75	82.35	82.35	73.68	70.00
Barren area	88.89	100.00	80.00	80.00	80.00	61.54
Water body	100.00	70.00	100.00	70.00	100.00	70.00
Built-up area	75.00	90.00	69.23	90.00	64.29	90.00

**Table 3:** Estimation of the kappa value.

Land cover	Kappa value	Asymp. Std. error^a^	Approx. *t*^b^	Approx. *P*
2000	0.83	0.050	15.218	0.00
2010	0.771937	0.055	14.491	0.00
2019	0.739364	0.056	14.258	0.00

^a^Not assuming the null hypothesis. ^b^Using the asymptotic standard error assuming the null hypothesis.

**Table 4:** Migration effect in land cover.

S. no.	Change matrix	Area (ha)
	Class	
1	Forest	5982.33
2	Others to forest	1629.72
3	Forest to others	1946.34
4	Shrub	218.79
5	Others to shrub	826.81
6	Shrub to others	284.76
7	Agriculture	7313.67
8	Other to agriculture	729.17
9	Agriculture to others	2064.3
10	Barren	7.65
11	Others to barren	750.42
12	Barren to others	453.95

**Table 5:** Coefficient of the model.

Model		Standardized coefficients	*t*	*P*
		*β*		
1	Constant	0.732	4.225	0
	Remittance	0.499	7.26	0
	Migrated members	0.331	4.822	0

Dependent variable: barren land				

**Table 6:** Regression coefficients of the variables.

Model		B	Coefficients^a^		*P*
			Beta	*t*	
1	Constant	207.306		15.525	0.00
	Dist_sett	0.131	0.333	8.366	0.00
	Dist_home	0.025	0.429	11.542	0.00
	Dist_water	−0.023	−0.158	−4.307	0.00
	Dist_forest	−0.584	−0.399	−9.863	0.00

a. Dependent Variable: Crop Damage (Amt._kg/Yr).

**Table 7:** Significance of the econometric model (ANOVA^a^).

S. no.	Model	*F*	*P*	*R*	*R* square
1.	Regression	130.751	0.000^b^	0.93	0.89

^a^Dependent variable, crop damage (Amt._kg/Yr). ^b^Predictors, constant, Dist_forest, Dist_water, Dist_home, Dist_sett.

## Data Availability

The data used to support the findings of this study are included within the article.
